# Burnout in Gastrointestinal Endoscopy

**DOI:** 10.1055/a-2932-0577

**Published:** 2026-10-15

**Authors:** Clara Benedetta Conti, Susanne M O’Reilly, Belen Martinez-Moreno, Irina Gabriela Diaconu, Didintle Mokgoko, Malina Wiesand, Preetha Thomas, Daniela Tabacelia, Jennifer Merten, Nada El-Domiaty, Morenike Osundina, Hadil Layka, Annalisa De Silvestri, Marianna Arvanitakis, Cesare Hassan, Amrita Sethi, Alessandro Repici, Roberta Maselli

**Affiliations:** 1 Interventional Endoscopy 9265 IRCCS San Gerardo dei Tintori Foundation HospitalMonzaLombardyItaly; 2 Centre for Colorectal DiseaseSt Vincent’s University HospitalDublinIreland; 3 Endoscopy Unit 16802 Hospital General Universitario Dr BalmisAlicanteValencian CommunitySpain; 4 Department for Gastroenterology and Interventional Endoscopy 60865 HELIOS Universitätsklinikum WuppertalWuppertalGermany; 5 Division of Gastroenterology, Department of Internal Medicine 705264 Charlotte Maxeke Johannesburg Academic HospitalJohannesburgSouth Africa; 6 Department of GastroenterologyClarunis University Digestive Health Care CenterbaselSwitzerland; 7 GastroenterologyMediclinic Kloof HospitalpretoriaSouth Africa; 8 Gastroenterology 434252 Elias Emergency University HospitalBucharestBucharestRomania; 9 Department of General, Visceral and Transplant SurgeryUniversity Hospital MuenstermunsterGermany; 10 Endemic Medicine Department 68900 Helwan UniversityHelwanCairo GovernorateEgypt; 11 Department of Medicine 107962 University College Hospital IbadanIbadanNigeria; 12 Department for Gastroenterology and Interventional Endoscopy 27664 HELIOS Hospital KrefeldKrefeldNRWGermany; 13 Clinical Epidemiology and Biometric UnitFoundation IRCCS Policlinico S. Matteo, PaviaPaviaItaly; 14 Gastroenterology 504992 Université Libre de Bruxelles - Campus ErasmeBruxellesBelgium; 15 Department of Biomedical Sciences 437807 Humanitas UniversityMilanItaly; 16 Endoscopy Unit 9268 IRCCS Humanitas Research HospitalRozzanoItaly; 17 Division of Digestive and Liver Disease 8645 Northwestern Medicine Central DuPage HospitalWinfieldIllinoisUnited States; 18 Department of Biomedical Sciences 437807 Humanitas UniversityPieve Emanuele, MilanSelezionaItaly; 19 Digestive Endoscopy Unit 9268 IRCCS Humanitas Research HospitalRozzanoItaly; 20 Endoscopic Unit, Department of Gastroenterology 9268 IRCCS Humanitas Research HospitalRozzanoItaly; 21 Department of Biomedical Sciences 150525 Humanitas UniversityRozzanoItaly

**Keywords:** other focus (of reviewers), epidemiology, quality and logistical aspects, training

## Abstract

**Objectives** Burnout is underinvestigated in endoscopy. Given the increasing complexity of endoscopic practice, understanding burnout is crucial to addressing the problem and finding possible risk and protective factors.

**Methods** A survey with 38 questions was internationally distributed via professional networks and social media. The survey investigated demographic data, professional experience, mentorship, work–life balance, and burnout. Burnout’ three symptoms were: feeling exhausted, feeling negative, and feeling emotionally drained. Individuals with all three symptoms were defined as burnout, whereas those having at least one symptom were defined as at risk of burnout. Univariate and multivariate statistical analyses were conducted using SPSS 30.0.

**Results** Totally, 553 endoscopists from 48 countries responded (51% male). Overall, 31% of the endoscopists were classified as having burnout, whereas 42% were considered at risk. At multivariable analysis, the delay of planning to have a family due to career was independently associated with burnout, OR: 2.48 [1.59–2.88]. Protective factors against burnout were: working in Asia 0.59 [0.38–0.93] and having a supportive mentor 0.50 [0.30–0.82].

**Conclusion** One out of every three endoscopists who responded to the questionnaire possessed features of burnout and one out of every two is at risk of developing it. Delay in family planning was associated with burnout, whereas working in Asia and having a supportive mentor were protective. This results may allow targeting of supportive programs to reduce burnout.

## Introduction

Burnout is an occupational phenomenon; however, a universally accepted definition is still lacking. In the 2021 consensus statement, an international expert panel proposed defining burnout as a condition characterized by physical and emotional exhaustion. 1 Conversely, the 11th revision of the International Classification of Diseases (ICD-11) describes burnout as a three-dimensional construct comprising feelings of exhaustion, negativism, and reduced professional effectiveness, with these dimensions being closely interrelated. 2 The Maslach Burnout Inventory (MBI) is a scale developed as a research instrument to assess burnout along the three dimensions. However, no universal criteria have been defined to diagnose burnout. 3 Exhaustion is considered its core, and some evidence suggests that it is clinically similar to depression. 4 5 6 A systematic review of 61 articles evaluated the association between burnout and psychological morbidity in physicians. While causality could not be definitively established due to the heterogeneity of the studies, physician burnout was found to be associated with depression, anxiety, and suicidality. 7 Importantly, occupational stress has been linked to coronary events, 8 severe fatigue, cognitive problems, absenteeism, and thoughts of quitting. 9 10 The Swedish Agency for Health Technology Assessment and Assessment of Social Services (SBU) found that work situations with limited opportunity combined with high demands, lack of supportive supervision, bullying, conflicts, perceptions of insufficient rewards, and job insecurity could increase the risk of developing depressive symptoms. 11 The SBU also found that people working in environments where both men and women feel they are treated fairly develop fewer symptoms of depression and exhaustion. 11

Burnout among physicians became more pronounced during and immediately after the COVID-19 pandemic (2020–2023). 12 13 Beyond the pandemic, burnout remains a relevant issue among physicians worldwide, 14 although it is still under-researched in the field of endoscopy. Ong et al. examined 11 studies about burnout among gastroenterologists. Work volume, age, and female gender were the three most frequently reported risk factors for increased levels of stress and burnout, mentioned in 72.7, 54.5, and 45.5% of studies, respectively. 15 Some other studies with small samples underlined that gastroenterologists who developed burnout were significantly younger ( *p* < 0.001) and had less leisure time ( *p* = 0.024). 16 17 18 Interestingly, stress and fatigue may affect even the quality of the clinical performance, as the effectiveness of endoscopic procedures in relation to temporal and organizational variables. Indeed, a study 19 found that colonoscopies performed in the afternoon negatively affected the adenoma detection rate, while an increased number of procedures was linked to a lower cecal intubation rate. 19 However, there are currently no large, international studies.

The primary aim of this study is to assess the prevalence of burnout among endoscopists and identify demographic, personal, and professional variables associated with it.

## Methods

A cross-sectional study was performed, through a survey made of 38 questions that was internationally distributed among endoscopists between October and November 2024. We disseminated it by using Irish society of gastroenterology (ISG) that invited 300 people, South African Gastroenterology Society (SAGES) that invited 126 people, Women in Endoscopy (WIE), that has more than 500 members, and social media ([in] LinkedIn, X (Twitter), [wa] WhatsApp). Social media are recognized as one of the most effective recruitment methods for observational studies. 20 21 According to the “impressions” officially recorded by [in] LinkedIn, the link to participate to the survey was seen by 4500 people only the first time that it was posted. At least 10 people reposted it, obtaining more visualizations. The link was also spread through X (Twitter), recording 4000 visualizations and via [wa] WhatsApp to almost 250 people. Therefore, we estimate that the survey reached more than 9500 people.

### Burnout Definition

Feeling exhausted, or worn out, was defined as physical strain related with the ergonomics of endoscopy combined with the cognitive saturation of high-volume, rapid-turnover lists. Feeling negative was defined as a dysfunctional coping mechanism where the physician creates psychological distance to manage stress, resulting in irritability or hostility to the work. Feeling emotionally drained by the job was defined as an indicator of emotional exhaustion, reflecting a crisis of confidence in which the endoscopist questions their professional competence or self-worth. For the purpose of this analysis, we defined people as at risk of burnout when at least one of the three was present and burnout when all three domains were present.

### Questionnaire

The questionnaire was anonymous, participation was voluntary, and no identifiable personal data were collected. Informed consent was obtained. The questions were designed to cover different aspects of both professional and personal life. We defined “dependants” as children or other people to care for. We defined “experienced endoscopists” as those with 10 or more years of activity and “advanced endoscopists” as the people performing hepatobiliary interventions (HPB) and/or advanced resections. We defined “hospitals with large volume of advanced endoscopic procedure” as those with at least 500 HPB and/or advanced resection cases per year. The three burnout key questions were: I feel exhausted (worn out) after work? I feel negative about my job? I feel emotionally drained by my job? The answers to these three questions were based on the Likert scale: all the time/most days/some days/occasionally/never. 22 For the purpose of this analysis, we defined as “yes” the answers all the time/most days/some days, whereas “no” the answers “occasionally/never.” In **Supplement Table 1** all the questions in detail are presented.

### Statistical Analysis

The data were exported from Survey monkey, collected, and analyzed for descriptive statistics and Chi square testing, using SPSS ver 30.0.0.0 software. Univariate binary logistic regression models were used to determine the association of individual variables with an endpoint using predefined categories. *P* value < 0.05 used to define significance. Stepwise multivariable logistic regression analysis was conducted to determine the most influential associated factors after adjusting for the other prespecified variables.

## Results

### Demographics

A total of 553 endoscopists participated in the survey (282/553, 51% male) from 48 different countries. The main demographic and professional data are presented in the Table 1 . Female representation within endoscopy units was reported to be limited. Specifically, 58% of respondents stated that female endoscopists comprised less than 25% of the workforce in their unit, while only 15% reported a female representation exceeding 50%.

**Table 1:** Demographic and professional characteristics of survey respondents ( *n* = 553).

Characteristic	Value (%)
Male	51
Asian/Asian American ethnicity	55.6
Caucasian ethnicity	31.5
Working in Asia	55
Working in America/Europe	39
Working in other countries	6
Leadership position	54
Experienced endoscopists	58
Early-career endoscopists	42
Trainees	10
Advanced endoscopists	56
Academic hospital practice	46
High-volume centers practice	30
Had a mentor (the mentor was helpful)	70 (86.7)
Have a partner	83.5
Have dependents	63
Delayed career progression due to family commitments	65 (46 women vs. 25 men)
Delayed family plans due to career progression	39 (51 women vs. 28 men)
Worked >40 h/week	79

### Feeling Exhausted (Worn Out)

A total of 336 respondents (61%) reported feeling exhausted after work. At multivariate analysis, feeling exhausted was significantly associated with delay in having a family due to career (70.6 vs. 39.4% OR: 1.7 [1.1–2.6]) and working more than 40 h per week (79 vs. 52.1%, OR: 1.3 [1.1–1.6]). Protective factors against feeling exhausted were: working in Asia (54.2 vs. 55.6%, OR: 0.29 [0.15–0.60]), being male (51 vs. 52.1% OR: 0.46 [0.30–0.71]), and having a helpful mentor (57.3 vs. 60.8%, OR: 0.48 [0.28–0.81]). Table 2 ; Fig. 1 .

**Table 2:** Variables significantly associated with feeling exhausted (worn out) at uni and multivariable analyses.

Variable	Univariable ( *p* value)	Multivariable ( *p* value)	OR [95% CI]
Male	**0.001**	**<0.001**	0.46 [0.30-0.71]
Working in Asia	**<0.001**	**<0.001**	0.29 [0.15-0.60]
Career commitments had delayed/held me back from having a family	**<0.001**	**<0.013**	1.7 [1.1-2.6]
Working hours per week > 40	**0.028**	**0.001**	1.3 [1.1-1.6]
Having a helpful mentor	**0.008**	**0.006**	0.48 [0.28-0.81]
Female	**0.001**		
Working in Europe/North America	**0.03**		
Working in other continents	0.24		
Being an experienced endoscopist	**<0.001**		
Being in a leadership role	0.28		
Having dependents	0.67		
Having a partner	0.19		
Having a mentor	0.63		
My family commitments had delayed my career	**0.012**		
Not able to maintain hobbies	**<0.001**		
Feeling that gender held me back in career progression	**<0.001**		
Being offered the same career opportunity as colleagues of the opposite gender	**0.003**		
Being offered the same career opportunity at the same time as colleagues of the opposite gender	**0.004**		
Don’t recommend a career in GI	0.05		

**Fig. 1:**
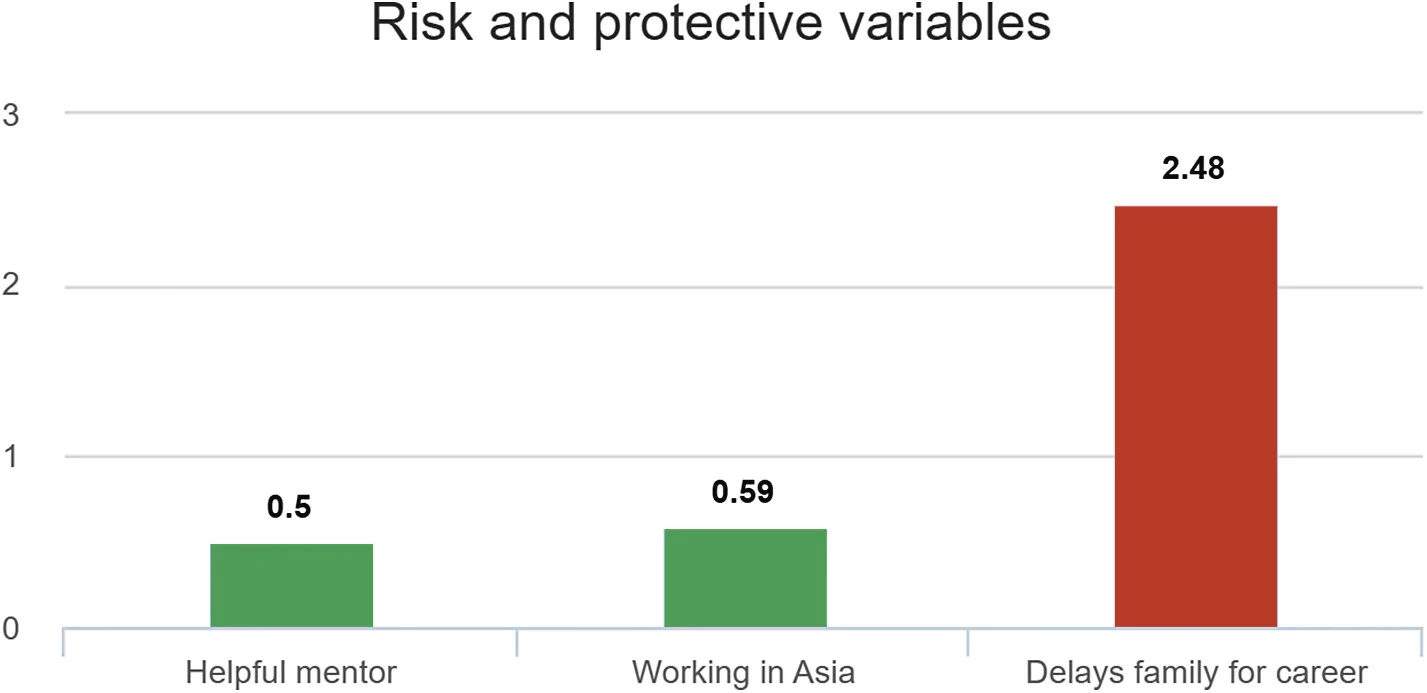
Variables independently associated to burnout. *X* -axis: Odds ratios; *y* -axis: variables associated to burnout. Green bars: reduced risk of burnout; red bar: higher risk of burnout.

### Feeling Negative

Overall, 232/553 (42%) of the responders declared to feel negative about their job. At multivariate analysis, feeling negative was related with delay in having a family due to career commitments (53.7 vs. 34.9%, OR: 2.33 [1.55–3.5]), whereas being experienced endoscopists and having helpful mentorship appeared to be protective factors: 33.9 versus 58.2%; OR: 0.75 [0.64–0.89] and 38.9 versus 60.8%, 0.48 [0.29–0.77], respectively ( Table 3 ; Fig. 1 ).

**Table 3:** Variables associated with feeling negative about the job at uni and multivariable analyses.

Variable	Univariable ( *p* value)	Multivariable ( *p* value)	OR [95% CI]
Experienced endoscopists	**0.001**	**<0.001**	0.75 [0.64-0.89]
Career commitments delayed/held me back from having a family	**<0.001**	**<0.001**	2.33 [1.55-3.5]
Having a helpful mentor	**0.01**	**0.002**	0.48 [0.29-0.77]
Working hours per week > 40	0.99		
Male	0.2		
Female	0.076		
Working in Europe/North America	**<0.001**		
Working in other continents	0.8		
Working in Asia	**<0.001**		
Being in a leadership role	**0.003**		
Having dependents	**0.032**		
Having a partner	0.36		
Having a mentor	0.89		
My family commitments had delayed career	**0.01**		
Not able to maintain hobbies	**<0.001**		
Feeling that gender held me back in career progression	**<0.001**		
Being offered the same career opportunity as colleagues of the opposite gender	**<0.001**		
Being offered the same career opportunity at the same time as colleagues of the opposite gender	**0.023**		
Don’t recommend a career in GI	**<0.001**		

### Feeling emotionally drained

A total of 271 (49%) of the responders declared to feel emotionally drained by their job. At multivariate analysis, the only variable associated with feeling emotionally drained was delay in having a family due to career (57.8 vs. 39.4%, 1.48 [1.04–2.13]). The protective factors against feeling emotionally drained were: being experienced endoscopists (39.8 vs. 58.1%, OR: 0.78 [0.66–0.91]) and working in Asia (40.9 vs. 55.6%, OR: 0.66 [0.45–0.96]). Table 4 , Fig. 1 .

**Table 4:** Variables associated with feeling emotionally exhausted at uni and multivariable analyses.

Variable	Univariable ( *p* value)	Multivariable ( *p* value)	OR [95% CI]
Experienced endoscopists	**0.001**	**0.002**	0.78 [0.66-0.91]
Career commitments delayed/held me back from having a family	**<0.001**	**0.001**	1.48 [1.04-2.13]
Working in Asia	**<0.001**	**0.029**	0.66 [0.45-0.96]
Working hours per week > 40	0.26		
Having a helpful mentor	**0.027**		
Female	**0.025**		
Working in Europe/North America	**<0.001**		
Working in other continents	0.11		
Male	0.23		
Being in a leadership role	0.19		
Having dependents	0.7		
Having a partner	0.09		
Having a mentor	0.84		
My family commitments had delayed my career	0.24		
Not able to maintain hobbies	**<0.001**		
Feeling that gender held me back in career progression	**0.015**		
Being offered the same career opportunity as colleagues of the opposite gender	**<0.001**		
Being offered the same career opportunity at the same time as colleagues of the opposite gender	**0.001**		
Don’t recommend a career in GI	**0.03**		

### Overall Burnout

Overall, the 42% of endoscopists presented at least one burnout symptom, the 34% two and the 31% all three burnout symptoms ( Fig. 2 ). At multivariate analysis, burnout was related to delays in having a family due to career (69.1 vs. 30.8%, OR: 2.48 [1.59–2.88]), whereas having a helpful mentor (31.3 vs. 68.6%, OR: 0.50 [0.30–0.82]) and working in Asia (30.8 vs. 69.1%, OR: 0.59 [0.38–0.93]) were statistically protective ( Table 5 )

**Fig. 2:**
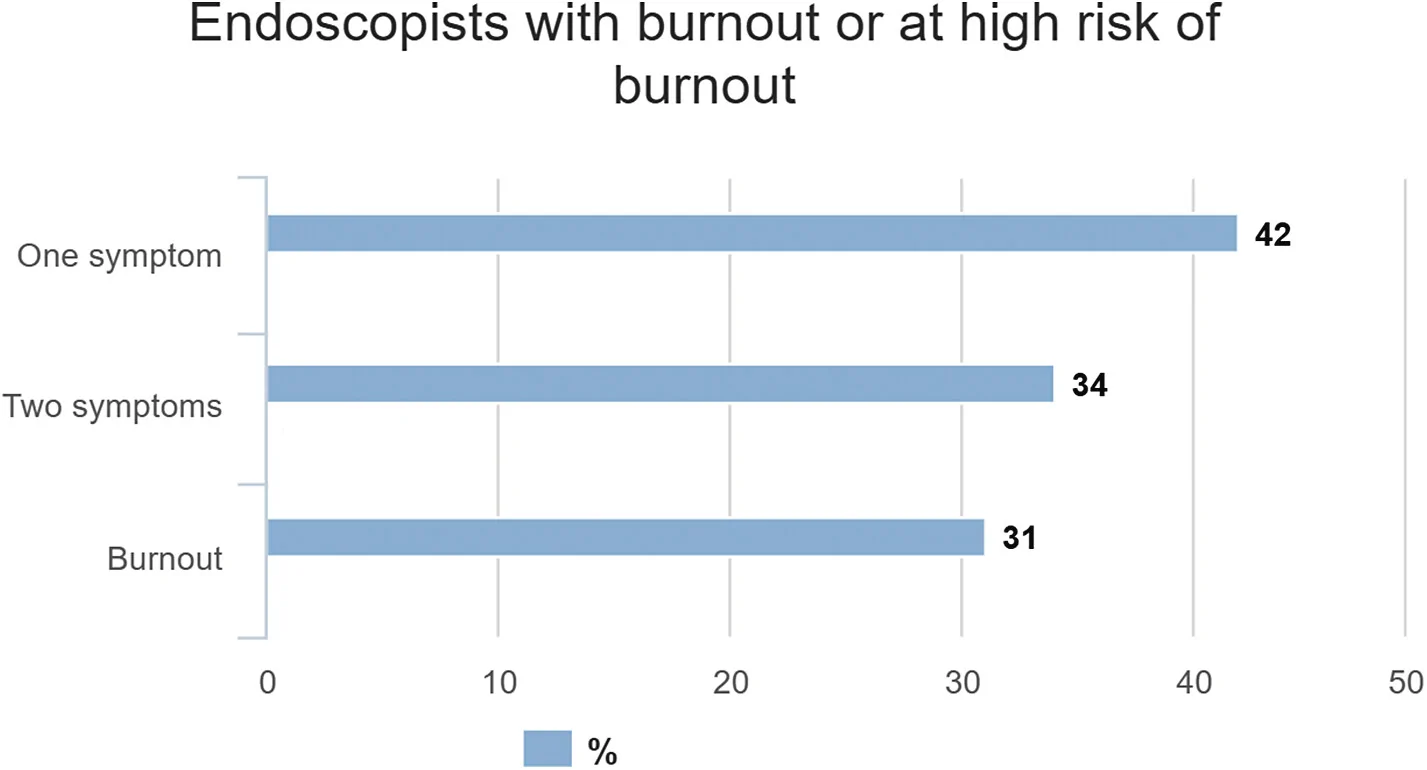
Percentage of endoscopists at risk or with burnout.

**Table 5:** Variables independently associated with burnout.

Variable	*p*	OR [95% CI]
My career commitments delayed/held me back from having a family	**<0.001**	2.48 [1.59-2.88]
The mentor had helped in advancing the career	**0.007**	0.50 [0.30-0.82]
Working in Asia	**0.002**	0.59 [0.38-0.93]

## Discussion

According to our data, burnout affects approximately one in three endoscopists, while one in two are at risk of developing it. These figures are substantial and comparable to those reported in surgical specialties, where burnout prevalence has been described as 42% among a small sample of orthopedic surgeons and 18% among surgical residents. 23 24 25 These findings are particularly relevant given that endoscopy is becoming increasingly interventional, with growing procedural complexity and a working environment that progressively resembles that of surgery. In addition, the increasing procedural volume and performance expectations may further contribute to psychological strain. This evolving scenario highlights the need for greater attention to occupational well-being within the field of endoscopy.

Secondly, we identified a key factor associated with burnout: the delay or postponement of planning to have a family due to career progression. This likely reflects limited free time, high workload, and demanding training pathways, which may interfere with personal life decisions. Notably, this association was independent of career hierarchy, as holding a leadership position was not linked to burnout, but groups at higher risk were early-career endoscopists and women. The latter finding aligns with existing evidence suggesting that career-related challenges in planning to have a family are more frequently reported by women, still underrepresented in advanced endoscopy. 26 Furthermore, early-career professionals may be more exposed to job insecurity and high performance pressure.

Third, we found protective factors against burnout. While cultural differences may contribute, workload patterns could also play a role: 60% of endoscopists in Asia reported working more than 60 h per week, compared to 83% in Europe and the United States. Additionally, positive mentorship was found to be a protective factor, consistent with findings among surgical residents. 22 Mentorship may provide both professional guidance and emotional support, improving resilience. In this context, the implementation of structured mentorship programs worldwide may represent a targeted strategy to mitigate burnout, as previously reported in the literature. 27 28 An example is the recently established Women in Endoscopy Mentorship Program promoted by Women In Endoscopy and Endoscopy on Air, 29 which aims to support career development and professional growth. Tailored mentorship initiatives can foster professional development, strengthen self-confidence, and facilitate the creation of supportive networks among peers and senior colleagues.

The main limitation of this study lies in the self-selected nature of the questionnaire responses, even if the anonymous design of the survey likely encouraged participants to answer honestly. Cultural differences may also have influenced the perception and reporting of burnout. Nevertheless, we believe that highlighting the global burden of burnout in endoscopy is an important first step toward conducting larger studies and address this issue.

## Conclusion

Our study underlines a high prevalence of burnout among endoscopists worldwide. We believe that the strength of our study lies in identifying the main variables positively and negatively related to burnout and the groups at higher risk, such as women and early-career endoscopists. These populations should be the focus of targeted strategies to reduce burnout, such as mentorship programs to create supportive networks, reduce the isolation, and enhance confidence.

## Supplementary Material

Ergänzendes Material Supplementary Material
