# Supplementary material for: Burnout in Gastrointestinal Endoscopy
Source: Endosc Int Open. 2026 Oct 15;14:a29320577. doi: 10.1055/a-2932-0577 (PMC13679639; doi:10.1055/a-2932-0577)
Supplement: Supplementary file 1 [file 10-1055-a-2932-0577_29777241.pdf]

Supplementary material.

Clara Benedetta Conti; Susanne M. O'Reilly; Belén Martínez Moreno; Irina Gabriela Diaconu; Didintle Mokgoko; Malina Wiesand; Preetha Thomas; Daniela Tabacelia; Jennifer Merten; Nada El-domiaty; Morenike Osundina; Hadil Layka; Annalisa De Silvestri; Marianna Arvanitakis; Cesare Hassan; Amrita Sethi; Alessandro Repici; Roberta Maselli

Burnout in Gastrointestinal Endoscopy

Supplement Table 1. Questionnaire

Theme	Question	Response Type
**Demographic Information**	1. What country are you from?	Single choice
	2. What country are you working in now?	Single choice
	3. What is your gender?	Single choice
	4. What is your ethnicity/race?	Single choice among a list
**Professional Experience**	5. How many years have you been performing endoscopy?	Single choice
	6. Are you an independent endoscopist?	Yes/No
	7. How many years since you completed training in diagnostic endoscopy?	Single choice
	8.*Have you had any breaks in your endoscopy training/performance?	Yes/No
	9. If yes, how long cumulatively have you spent out of a post where you were scoping regularly?	Single choice
	10. Are you currently or have you previously been in a leadership role in your department?	Yes/No
	11. Do you have any dependents?	Yes/No
	12.*Do you currently have a partner?	Yes/No
	13. If you currently have a partner, are they in full-time employment?	Yes/No
**Training**	14. Does your unit have a structured advanced endoscopy training program?	Yes/No
	15. Which procedures do you perform?	Checkboxes
	16. Are you an independent advanced endoscopist in the procedures you have selected above?	Yes/No
**Institution type**	17.*Are there endoscopy fellows in your center?	Yes/No
	18. What volume of HPB (hepato-biliary) endoscopy cases does your center perform each year?	Single choice among the list
	19. What volume of non-HPB advanced endoscopy cases does your center perform each year?	Single choice
**Mentorship**	20. Have you had a mentor in advanced endoscopy?	Yes/No
	21. Was/Is your mentor male or female?	Gender
	22. Did you experience differences between a male and a female mentor? Provide examples below.	Open text
	23. Has your mentor helped advance your career, in your opinion?	Yes/No
**Work-Life Balance**	24. Have family commitments held you back or delayed your career progression?	Yes/No

Theme	Question	Response Type
	25. Have your career commitments delayed you in having a family?	Yes/No
	26. What type of hospital are you primarily based in?	Single choice among a list
	27. Is your institution active in clinical/academic research in gastroenterology?	Yes/No
	28. Who performs general endoscopy in your unit?	Checkboxes
	29. What percentage, roughly, of endoscopists in your unit are female?	Single choice
	30. How many hours do you work per week?	Single choice
**Burnout**	31. I feel worn out	Likert scale
	32. I feel negative about my work	Likert scale
	33. I feel emotionally drained by my work	Likert scale
	34. I am able to maintain hobbies outside of work	Yes / No / Sometimes
**Gender Issues**	35. In your opinion, has your gender held you back in developing your advanced endoscopy career or in your department?	Yes / No / Maybe
	36. Do you think you are offered the same career development opportunities as male/female colleagues?	Yes / No
	37. Do you think you are offered career development opportunities at the same time as male/female colleagues?	Yes / No
	38. Would you recommend a career in GI to other male/female physicians?	Yes / No

*=optional questions. HPB: hepatobiliary. Question 4. Single choice among the list: American Indian/Alaska native; Asian/Asian American; Black/African American; Hispanic/Latino; Middle Eastern/North African; Hawaiian/other Pacific Islander; Caucasian; Other. Question 15. Checkboxes: diagnostic EUS; Therapeutic EUS; Basic ERCP; Complex ERCP; Endoscopic submucosal resection; Lower GI third space endoscopy; Upper GI third space endoscopy. Question 18. Single choice among the list: < 100/100-300/300-500/500-1000/>1000. Question 26. Single choice among the list: Primary/Secondary/Tertiary. Question 28. Checkboxes: gastroenterologists/Surgeons/Nurses. Question 31, 32, 33. Likert scale: all the time/most days/some days/occasionally/never.
